# Choice of Cell-Delivery Route for Skeletal Myoblast Transplantation for Treating Post-Infarction Chronic Heart Failure in Rat

**DOI:** 10.1371/journal.pone.0003071

**Published:** 2008-08-27

**Authors:** Satsuki Fukushima, Steven R. Coppen, Joon Lee, Kenichi Yamahara, Leanne E. Felkin, Cesare M. N. Terracciano, Paul J. R. Barton, Magdi H. Yacoub, Ken Suzuki

**Affiliations:** 1 Harefield Heart Science Centre, National Heart & Lung Institute, Imperial College London, Harefield, Middlesex, United Kingdom; 2 Translational Cardiovascular Therapeutics, William Harvey Research Institute, Barts and The London, Queen Mary's School of Medicine and Dentistry, London, United Kingdom; Baylor College of Medicine, United States of America

## Abstract

**Background:**

Intramyocardial injection of skeletal myoblasts (SMB) has been shown to be a promising strategy for treating post-infarction chronic heart failure. However, insufficient therapeutic benefit and occurrence of ventricular arrhythmias are concerns. We hypothesised that the use of a retrograde intracoronary route for SMB-delivery might favourably alter the behaviour of the grafted SMB, consequently modulating the therapeutic effects and arrhythmogenicity.

**Methods and Results:**

Three weeks after coronary artery ligation in female wild-type rats, 5×10^6^ GFP-expressing SMB or PBS only (control) were injected *via* either the intramyocardial or retrograde intracoronary routes. Injection of SMB *via* either route similarly improved cardiac performance and physical activity, associated with reduced cardiomyocyte-hypertrophy and fibrosis. Grafted SMB *via* either route were only present in low numbers in the myocardium, analysed by real-time PCR for the Y-chromosome specific gene, *Sry*. Cardiomyogenic differentiation of grafted SMB was extremely rare. Continuous ECG monitoring by telemetry revealed that only intramyocardial injection of SMB produced spontaneous ventricular tachycardia up to 14 days, associated with local myocardial heterogeneity generated by clusters of injected SMB and accumulated inflammatory cells. A small number of ventricular premature contractions with latent ventricular tachycardia were detected in the late-phase of SMB injection regardless of the injection-route.

**Conclusion:**

Retrograde intracoronary injection of SMB provided significant therapeutic benefits with attenuated early-phase arrhythmogenicity in treating ischaemic cardiomyopathy, indicating the promising utility of this route for SMB-delivery. Late-phase arrhythmogenicity remains a concern, regardless of the delivery route.

## Introduction

Both experimental and initial clinical studies have shown that transplantation of skeletal myoblasts (SMB) into the heart is a promising treatment for post myocardial infarction (MI) chronic heart failure (HF). However, a recent large-scale randomised clinical study has suggested that the therapeutic efficacy of this strategy may not be as substantial as expected when conjugated with coronary artery bypass surgery [1], [2]. In addition, the risk of arrhythmia occurrence associated with this treatment is not fully understood [1]–[4]. It is therefore essential to explore a strategy to amplify the therapeutic benefits and reduce the arrhythmogenicity for the future success of SMB transplantation.

Of note, almost all experimental and clinical studies to date have utilised intramyocardial (IM) injection for SMB delivery into the heart. However, this method is known to have disadvantages including formation of islet-like cell-clusters and induction of myocardial damage and disruption [5], [6]. We hypothesised that these adverse events may be a cause of the insufficient therapeutic benefits and arrhythmia occurrence following the treatment. In contrast, intracoronary (IC) injection has been reported to enable widespread dissemination of donor cells with less myocardial damage [6]–[8]. Although the antegrade IC route is frequently used in clinical trials of bone marrow cell injection into the heart [9], this route is likely to carry a high risk of coronary embolism when SMB are injected because of their larger cell-size [7]. On the other hand, retrograde IC injection (injection into the cardiac veins) has been demonstrated to achieve widespread dissemination of donor cells in the heart with little risk of coronary embolism [8]. Cells disseminated cells in such a gentle manner are more likely to survive and maintain important properties such as differentiation, integration and secretion, consequently leading to an enhanced therapeutic outcome and reduced arrhythmogenicity.

In this study, we therefore examined the efficiency and pattern of cell-distribution, behaviour of the grafted cells, therapeutic efficacy and arrhythmogenicity after retrograde IC injection of SMB into the post-MI chronically failing heart, in comparison with direct IM injection.

## Methods

The investigation conforms to the *Guide for the Care and Use of Laboratory Animals* published by the US National Institutes of Health (NIH Publication No. 85-23, revised 1996). All surgical procedures and evaluations, particularly cardiac function measurement by echocardiography, were carried out in a blinded manner.

### Collection, culture and characterisation of SMB

Primary SMB were isolated from male GFP-transgenic Sprague-Dawley (SD) rats (200–250 g, Rat Research & Resource Center) by the single fibre method (*n* = 10) [10]. Briefly, the extensor digitorum longus was digested with 0.2% type I collagenase (Sigma) and individual, intact myofibres were then placed onto a culture plate, from which SMB migrated and proliferated in growth medium containing 20% foetal calf serum, 10% horse serum, 0.5% chick embryo extract and 5 ng/ml basic fibroblast growth factor (Molecular Probes) in DMEM with 1% penicillin/streptomycin. Myogenic differentiation ability of the cells was confirmed to be the same as that of SMB derived from wild-type rats by culturing in low serum (2% horse serum) medium [10].

### Generation of post-MI chronic HF and SMB injection

Female wild-type SD rats (150–200 g, Harlan) underwent permanent left coronary artery (LCA) ligation under mechanical ventilation and 1.5% isoflurane inhalation (*n* = 190) [6]. At 3 weeks after LCA ligation, cell injection *via* either the direct IM or retrograde IC route was carried out (*n* = 165) [6]. For direct IM injection, a total of 5×10^6^ SMB suspended in 100 µl of PBS (SMB-IM group, *n* = 48) or 100 µl of PBS (PBS-IM group, *n* = 32) was injected into two sites of the peri-infarct area using a 31-gauge needle. For retrograde IC injection, 5×10^6^ SMB suspended in 500 µl of PBS (SMB-IC group, *n* = 49) or 500 µl of PBS (PBS-IC group, *n* = 36) were slowly injected with a constant pressure over 30 seconds through a 24-gauge catheter (BD Biosciences) inserted into the left cardiac vein. The stem of left cardiac vein was snared from the start of injection till 30 seconds after the completion of injection.

### Assessment of cardiac function and structure

Echocardiography (Sequoia 512 and 15-MHz probe, Siemens Medical) was carried out under 1.5% isoflurane inhalation *via* a nose corn at one day before, and days 3, 7, 28 and 84 after injection (*n* = 10 in each group at each time point) [6]. Left ventricular ejection fraction (LVEF) was assessed by 2-dimensional mode at the mid-papillary level, and LV diastolic/systolic dimensions (LVDd/LVDs) were assessed by M-mode [11]. Transmitral E/A flow ratio was assessed by spectral Doppler traces [12].

### Assessment of spontaneous arrhythmia and physical activity by telemetry

Spontaneous arrhythmia occurrence and physical activity were assessed by telemetry (Data Sciences International) [13], [14]. A transmitter and two electrodes were implanted 1 day before injection. ECG and signal strength were continuously monitored at day 1, 3, 7, 14, 28, 42, 56 and 84 (*n* = 7 in the PBS-IM and PBS-IC groups and *n* = 8 in the SMB-IM and SMB-IC groups). The hourly number of ventricular premature contractions (VPC), calculated as an average over 24-hours recording, and frequency of VT were assessed.

The signal strength, which is changed by physical movement of the rat, was monitored at 64 Hz as an indicator of physical activity [14]. The average of 24 hours recording was calculated using software (DSI Dataquest A.R.T. ™ Analysis, Data Science International). Low counts of change in signal strength indicate reduced physical activity.

### Arrhythmia induction by isoproterenol administration

Isolated hearts were perfused at 100 cm H_2_O with modified Krebs-Henseleit buffer (144.9 mM Na^+^, 126.3 mM Cl^−^, 4.9 mM K^+^, 25.0 mM HCO_3_^−^, 1.2 mM SO_4_^−^, 1.2 mM Mg^2+^, 1.25 mM Ca^2+^, 1.2 mM PO_4_^3−^ and 10.0 mM glucose; gassed with 95% O_2_+5% CO_2_ at 37°C) using a Langendorff apparatus at day 84 (*n* = 3 in the PBS-IM and PBS-IC groups and *n* = 6 in the SMB-IM and SMB-IC groups) [7]. After 20-minutes stabilisation, 1×10^−7^ M isoproterenol (Sigma) was administered continuously *via* the perfusate for 10 minutes [15] with the ECG being monitored for the analysis using MP35 and Biopac Systems (Linton Instrumentation).

### Histological analysis

Hearts were collected at day 3 and 28, fixed by 4% paraformaldehyde perfusion, cut transversely and frozen (*n* = 5 in each group). Fifteen-µm cryosections were labelled with polyclonal anti-GFP (1∶1000 dilution, Molecular Probes) [6], monoclonal anti-CD45 (1∶250 dilution, BD Pharmingen) or monoclonal anti-connexin(Cx)43 (1∶500 dilution, Chemicon) [16] antibodies. The labelled sections were visualised by either HRP-based kit (DAKO) with haematoxylin counterstaining or by immunoconfocal microscopy (LSM510, Zeiss).

To evaluate myocardial collagen deposition, cryosections from day-28 hearts were stained with 0.1% Picrosirus red (*n* = 5 in each group) [17]. The collagen volume fraction was semi-quantitatively assessed using NIH image-analysis software (randomly selected 20 high-power fields per one heart). To evaluate myocardial capillary density, cryosections at day 28 were labelled with polyclonal anti-von Willebrand Factor (vWF) antibody (1∶250 dilution, DAKO) and assessed using confocal microscopy (*n* = 5 in each group) [18], [19]. The number of capillary vessels, which were positively stained and had 5–10 µm in diameter, was counted in the peri-infarct area (randomly selected 16 high-power fields per one heart) and was averaged to express a capillary density (per 1 mm^2^).

### Quantitative analysis of the graft survival by real-time PCR for Sry

The presence of male grafted cells in the female heart was quantitatively assessed to define graft survival by using real-time PCR (ABI PRISM 7700 and TaqMan chemistry) for the Y-chromosome specific *Sry* gene in DNA extracted from the entire LV walls. *Sry* levels were normalised to the DNA amount using the autosomal single copy gene, *oesteopontin* [6]. Data were analysed using the Ct method [20].

A standard curve was prepared from serial dilution series (1, 1/3, 1/9, 1/27, 1/81 and 1/243) of the DNA extracted from a mixture of male SMB (5×10^6^) and female entire LV walls at 21 days after LCA ligation (*n* = 3). The number of surviving grafted cells at day 3, 7, 28 and 84 (*n* = 5 in the SMB-IM and SMB-IC groups at each time point) was estimated by correcting relative *Sry* expression using this standard curve.

### Cell area planimetry of isolated native cardiomyocytes

At day 28, cardiomyocytes were isolated from the heart by enzymatic dissociation (*n* = 6 in the normal female SD rats, PBS-IM, SMB-IM and SMB-IC group) [21]. After perfusion with collagenase (1.3 mg/mL, Worthington Biochemical) and hyaluronidase (0.6 mg/mL, Sigma), the LV tissue was cut into small pieces and further dissociated to yield a single-cell suspension. The projected 2-dimensional area for each GFP-negative rod-shape cardiomyocyte was assessed by NIH image-analysis software. The cell number examined was 221 in the normal, 225 in the PBS-IM, 328 in the SMB-IM and 336 in the SMB-IC groups.

### Statistical analysis

All values are expressed as Mean±SEM. HR, LVDd/Ds, peak E/A, LVEF, physical activity and VPC number were compared using 2-way ANOVA followed by Bonferroni's test for individual significant difference. Collagen volume, capillary density and cardiomyocyte-area were compared using 1-way ANOVA followed by Bonferroni's test. Frequency of spontaneous and induced VT was compared using chi square test. HR and VPC number under isoproterenol were compared using paired-t test. *p*<0.05 was considered to be statistically significant.

## Results

### Mortality after LCA ligation and cell injection

Mortality after LCA ligation before cell injection was 11.6% (21/190) in total. Among the surviving rats, 4 (2.6%) were excluded due to their LVEF being more than 40% one day before cell injection. Mortality after cell injection was similar in all groups; 6.3% (2/32) in the PBS-IM, 8.3% (3/36) in the PBS-IC, 8.3% (4/48) in the SMB-IM, 8.2% (4/49) in the SMB-IC groups.

### Improved cardiac function and physical activity after cell injection

Cardiac function and dimensions were serially assessed using echocardiography. Baseline values (normal rats) were 363±18 bpm for HR, 73.9±1.8% for LVEF, 6.7±0.3/3.8±0.3 mm for LVDd/LVDs and 1.7±0.2 for peak E/A (*n* = 10). At 20 days after LCA ligation, reduced LVEF (32.9±0.2%, *p*<0.001), enlarged LVDd/LVDs (9.0±0.1/7.7±0.2 mm, *p*<0.001) and reduced E/A (1.2±0.1 *p*<0.01) were observed (*n* = 165).

**Figure 1:**
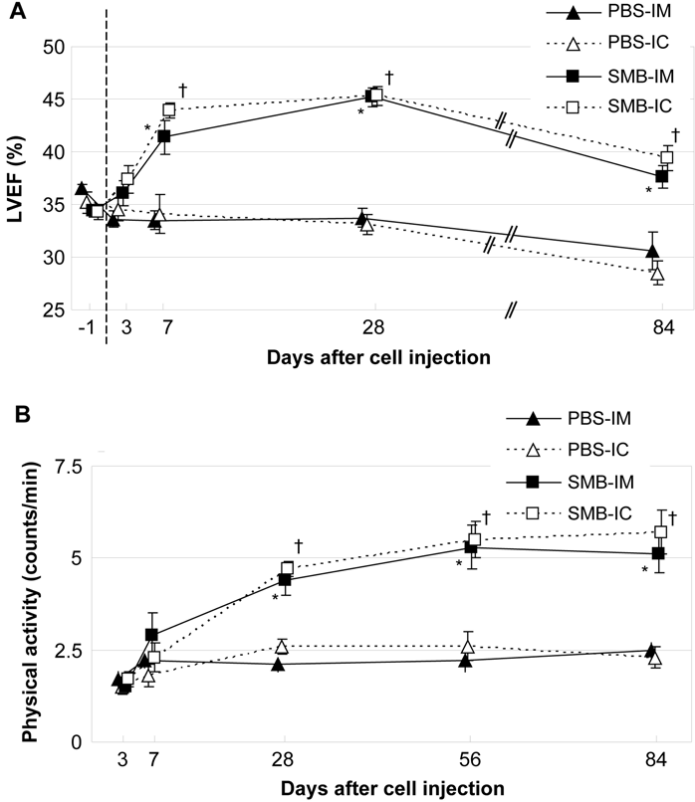
Cardiac function and physical activity. A: LVEF, assessed by 2-dimensional mode echocardiography, was significantly greater in the SMB-IM and SMB-IC groups than that in the PBS-IM and PBS-IC groups, respectively, at day 7, 28 and 84. *n* = 10 in each group at each time point. −1; one day before injection. B: Physical activity in the SMB-IM and SMB-IC groups was greater than that in the PBS-IM and PBS-IC groups at day 28 and 84. *n* = 7 in the PBS-IM and PBS-IC groups; *n* = 8 in the SMB-IM and SMB-IC groups at each time point. *p<0.05 *vs.* the PBS-IM group; ^†^ *p*<0.05 *vs.* the PBS-IC group at each time point.

LVEF after PBS injection *via* either route was not significantly changed throughout the experimental period (Figure 1A) but was significantly greater following injection of SMB *via* either route between day 7 and 84 (*p*<0.001). Compared to before injection (33.4±0.6% in the SMB-IM and 34.5±0.6% in the SMB-IC groups, respectively), LVEF was improved by day 7 after IM or IC SMB injection (43.6±0.7% and 43.9±0.7%, respectively, *p*<0.05). SMB injection *via* either route resulted in lower HR, smaller LVDs and greater E/A at day 28, but not at day 84, compared to PBS injection (Table 1).

**Table 1:** Cardiac performance after SMB transplantation.

	Before				Day 28				Day 84			
	HR (bpm)	LVDd (mm)	LVDs (mm)	Peak E/A	HR (bpm)	LVDd (mm)	LVDs (mm)	Peak E/A	HR (bpm)	LVDd (mm)	LVDs (mm)	Peak E/A
**PBS-IM**	373±20	8.8±0.2	7.3±0.2	1.3±0.2	406±12	10.0±0.2	8.7±0.2	0.7±0.1	431±43	9.7±0.2	8.9±0.4	0.8±0.0
**PBS-IC**	370±23	8.7±0.1	7.4±0.2	1.4±0.2	401±11	9.5±0.2	8.8±0.2	0.8±0.1	432±29	10.0±0.4	8.7±0.5	0.8±0.1
**SMB-IM**	370±10	9.0±0.1	7.5±0.1	1.3±0.1	354±9*	9.9±0.2	8.0±0.2*	1.8±0.1*	372±12	10.6±0.4	9.0±0.4	1.3±0.2
**SMB-IC**	366±9	9.1±0.1	7.7±0.1	1.3±0.1	356±7†	9.6±0.2	7.7±0.3†	1.5±0.3†	372±7	10.4±0.4	9.3±0.6	0.9±0.2

*n* = 10 in each group at each time point.

* *p*<0.05 *vs.* PBS-IM.

† *p*<0.05 *vs.* PBS-IC.

All the groups showed an improvement in physical activity by day 7, though this was not statistically significant, presumably due to recovery from the surgical stress. Whereas physical activity did not increase day 7 after PBS injection *via* either route, injection of SMB *via* either route improved physical activity by day 28 (Figure 1B). Improved activity after SMB injection continued until day 84.

### Occurrence of spontaneous arrhythmias following SMB injection

**Figure 2:**
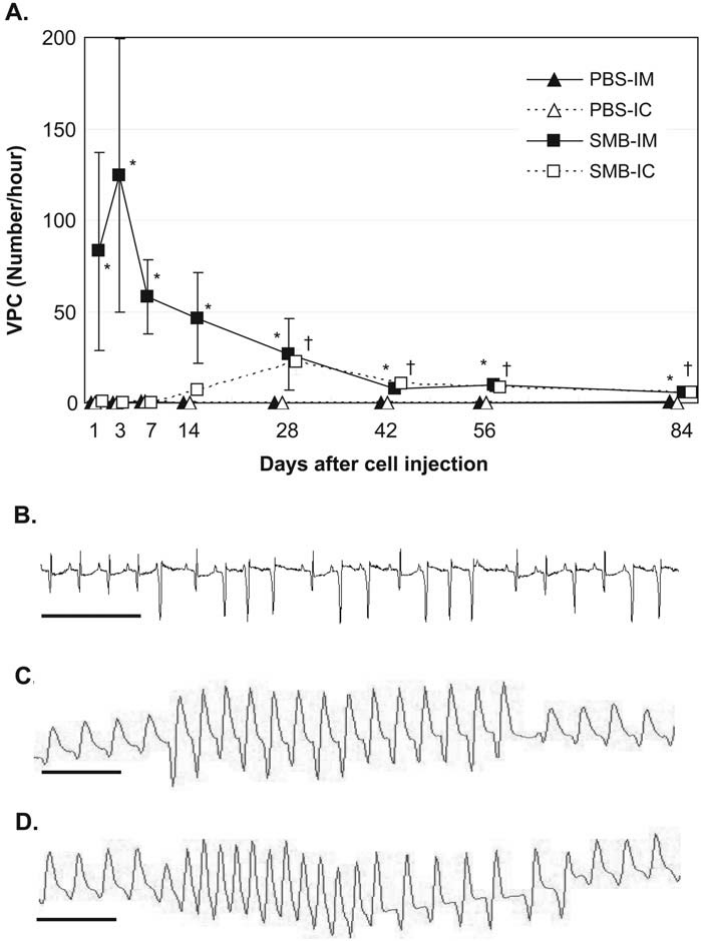
Spontaneous and induced occurrence of arrhythmias. A: The hourly number of spontaneous VPC was calculated as an average of the VPC number detected over the 24-hour continuous ECG recording by telemetry. VPC were frequently detected at day 1 only in the SMB-IM group with a peak at day 3. The VPC number in the SMB-IM group decreased by day 14, but a small number of VPC were persistent until day 84. In contrast, in the SMB-IC group, VPC rarely occurred until day 7, while a small number of VPC were observed between day 14 and 84. Both the PBS-IM or PBS-IC groups rarely showed VPC throughout the study. *n* = 7 in the PBS-IM and PBS-IC groups; *n* = 8 in the SMB-IM and SMB-IC groups at each time point. *p<0.05 *vs.* the PBS-IM group; ^†^ *p*<0.05 *vs.* the PBS-IC group at each time point. B: Representative ECG of spontaneous VT in the SMB-IM group. C and D: Representative ECGs of induced VT by isoproterenol administration under Langendorff isolated-heart perfusion in the SMB-IM (C) and SMB-IC groups (D). Scale bar = 500 msec.

Spontaneous arrhythmias were assessed by continuous ECG monitoring using telemetry. Arrhythmias rarely occurred 1 day before cell transplantation (20 days after permanent LCA ligation) or till 84 days after PBS injection *via* either route (Figure 2A). Of note, IM injection of SMB caused frequent VPC (≥40/hour) within 24 hours after injection, which lasted until day 14 with a peak at day 3 (124±75/hour). In addition, only IM injection of SMB caused VT in 50% of animals at day 3 and day 7 (Figure 2B), whereas there was no animal showing VT in the other groups at the same time points. Subsequently, the number of VPC gradually decreased, although a small number of VPC (≥5/hour) persistently remained until day 84. In contrast, in the SMB-IC groups, VPC or VT rarely occurred until day 7, whereas a small number of VPC occurred at day 14 and persisted to day 84. No spontaneous VT was observed at day 84 in any group.

### Isoproterenol-induced arrhythmia

Isoproterenol was administered to the hearts under Langendorff perfusion in order to uncover latent arrhythmogenicity. Isoproterenol administration increased HR in all groups (*p*<0.05, Table 2). The frequency of VPC was increased by isoproterenol administration in the hearts after SMB injection *via* either route (*p*<0.05), while VPC number was not significantly increased in the hearts after PBS injection. Of note, VT was induced in 83.3% hearts after SMB injection *via* either route (Figure 2C and D), while no VT was induced in the hearts after PBS injection.

**Table 2:** Isoproterenol-induced arrhythmias.

	Heart rate (bpm)		Number of VPC (/hour)		Samples showing VT (%)	
	Baseline	Isoproterenol	Baseline	Isoproterenol	Baseline	Isoproterenol
**PBS-IM**	235±29	375±17*	2±2	4±2	0.0%	0.0%
**PBS-IC**	260±12	353±18*	0±0	16±13	0.0%	0.0%
**SMB-IM**	263±8	380±21*	28±9	87±43*	0.0%	83.3%†
**SMB-IC**	224±28	367±24*	4±2	65±23*	0.0%	83.3%‡

*n* = 7 in the PBS-IM and PBS-IC groups; *n* = 8 in the SMB-IM and SMB-IC groups.

* *p*<0.05 *vs.* baseline value.

† *p*<0.05 *vs.* the PBS-IM group.

‡ *p*<0.05 *vs.* the PBS-IC group.

### Behaviour of grafted cells in the myocardium

**Figure 3:**
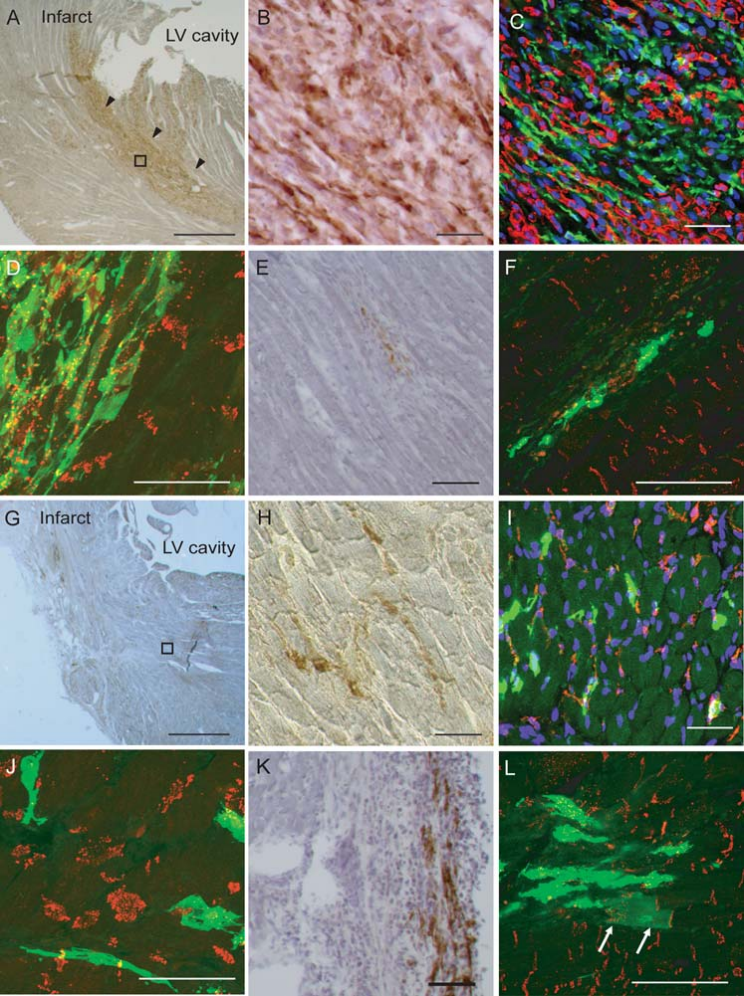
Behaviour of grafted cells in the myocardium. A: Immunohistolabelling for GFP with haematoxylin counterstaining detected GFP-positive (brown) cell-clusters disrupting the myocardial structure in the SMB-IM group at day 3. B: In higher magnification of the outlined area in A, the cell-clusters consisted of GFP-positive and negative cells. C: Confocal micrograph (green for GFP, red for CD45, blue for nuclei) in the SMB-IM group at day 3 shows that GFP-positive cells are surrounded by a number of GFP-negative CD45-positive cells. D: Confocal micrograph (green for GFP, red for Cx43) in the SMB-IM group at day 3 shows the lack of gap junctions between GFP-positive cells and adjacent GFP-negative cardiomyocytes. E: At day 28 in the SMB-IM group, a small number of GFP-positive cells are surrounded by fibrous tissues. F: Confocal micrograph (green for GFP, red for Cx43) in the SMB-IM group at day 28 shows no gap-junctions between the GFP-positive cells and adjacent GFP-negative cardiomyocytes. G: At day 3 in the SMB-IC group, GFP-positive cells were widely disseminated without disrupting the myocardial structure. Please note that it is difficult to see individual disseminated cells in the low magnification image. H: Higher magnification of the outlined areas in G shows GFP-positive cells with less myocardial damage and inflammation, compared to the SMB-IM group. I: Confocal micrograph (green for GFP, red for CD45, blue for nuclei) shows less accumulation of CD45-positive cells surrounding the GFP-positive cells compared to the SMB-IM group. J: Confocal micrograph (green for GFP, red for Cx43) in the SMB-IC group at day 3 does not show any gap-junctions between the GFP-positive cells and cardiomyocytes. K: At day 28 in the SMB-IC group, a small number of GFP-positive cells were detected. L: Confocal micrograph (green for GFP, red for Cx43) shows the only example of a GFP-positive cell with cardiomyocyte-like morphology forming gap-junctions with a native cardiomyocyte (arrows) in the SMB-IC group. Scale bar = 500 µm in A and F, 50 µm in B–E and G–L.

Grafted cells were identified in the myocardium by labelling for GFP. At day 3 after IM injection of SMB, islet-like GFP-positive cell-clusters were formed which disrupted the myocardial structure in the peri-infarct area (Figure 3A). These cell-clusters consisted of grafted GFP-positive cells and native GFP-negative CD45-positive cells (Figure 3B and C). Formation of Cx43-gap junctions was not evident between the grafted cells and surrounding native cardiomyocytes (Figure 3D). At day 28 after IM injection of SMB, the size of the GFP-positive grafts had generally decreased (Figure 3E) and the majority of the GFP-positive cells appeared to be isolated from the native cardiomyocytes (Figure 3F).

In contrast, at day 3 after retrograde IC injection of SMB, myocardial structure was not disrupted by GFP-positive cell-clusters (Figure 3G) and GFP-positive cells were disseminated among the native cardiomyocytes in peri-infarct areas (Figure 3H). The degree of CD45-positive cell-accumulation surrounding the GFP-positive cells was lower than that after IM injection of SMB (Figure 3I). Although the grafted cells in the peri-infarct area appeared to have a direct contact with native cardiomyocytes, formation of Cx43-gap junctions between these cells was not evident (Figure 3J). There was no observation suggesting coronary embolism after retrograde IC injection of SMB. At day 28 after retrograde IC injection, GFP-positive cells were detected with an elongated myotube-like morphology (Figure 3K).

The frequency of GFP-positive cardiomyocyte-like cells derived from transdifferentiation or fusion of donor SMB was extremely low. Our thorough examination detected only one such cell after retrograde IC injection of SMB (Figure 3L). This GFP-positive cell with cardiomyocyte-morphology formed Cx43-gap junctions with GFP-negative native cardiomyocytes.

### Poor graft survival after SMB injection

**Figure 4:**
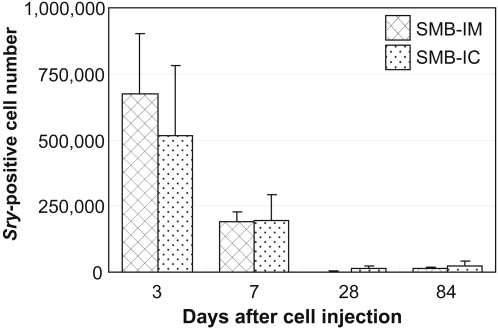
Survival of grafted cells in the myocardium. Time-course of donor-derived cell number present in the native LV was estimated by real-time PCR for the Y-chromosome specific gene, *Sry*. Both the SMB-IM and SMB-IC groups showed a small number of donor-derived cells at day 3, which further decreased by day 28. *n* = 5 in each group at each time point.

In our model, male cells were injected into female hearts in order to quantitatively analyse graft survival using real-time PCR for the Y-chromosome specific gene, *Sry*. At day 3 after IM injection of SMB, 6.7±2.3×10^5^ male cells (14±5% of total injected cells) were present in the LV, while 5.2±2.7×10^5^ cells (10±5%) existed at day 3 after retrograde IC injection of SMB (Figure 4). The number of grafted cells present in the LV decreased to 1.9±0.4×10^5^ cells (4±1%) or 2.0±1.0×10^5^ cells (4±1%) by day 7 and further decreased to 0.2±0.1×10^5^ cells (0.3±0.2%) or 0.2±0.1×10^5^ cells (0.3±0.2%) by day 28 after direct IM or retrograde IC injection of SMB, respectively.

### Changes in the native myocardium after SMB injection

**Figure 5:**
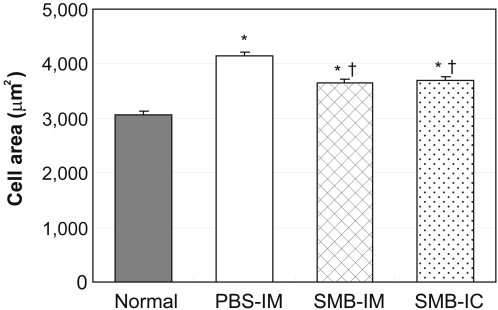
Size of native cardiomyocytes. The size of native cardiomyocytes, isolated by enzymatic digestion at day 28 after cell transplantation, was assessed. Cardiomyocytes in the SMB-IM and SMB-IC groups were significantly smaller than those in the PBS-IM group, but still larger than those from the normal hearts. *n* = 5 in the PBS-IM, SMB-IM and SMB-IC groups; *n* = 6 in the Normal group. *p<0.05 *vs.* the Normal group; ^†^ *p*<0.05 *vs.* the PBS-IM group.

Native (GFP-negative) cardiomyocytes, isolated by enzymatic dissociation, were assessed for cell size. Cardiomyocytes after PBS injection were significantly larger than those from uninfarcted, normal animals (4,143±68 µm^2^ *vs.* 3,074±61 µm^2^, *p*<0.05, Figure 5). This hypertrophy showed a significant, but not complete, regression in the cardiomyocytes isolated after either IM injection of SMB (3,657±53 µm^2^) or retrograde IC injection of SMB (3,690±64 µm^2^, *p*<0.05).

**Figure 6:**
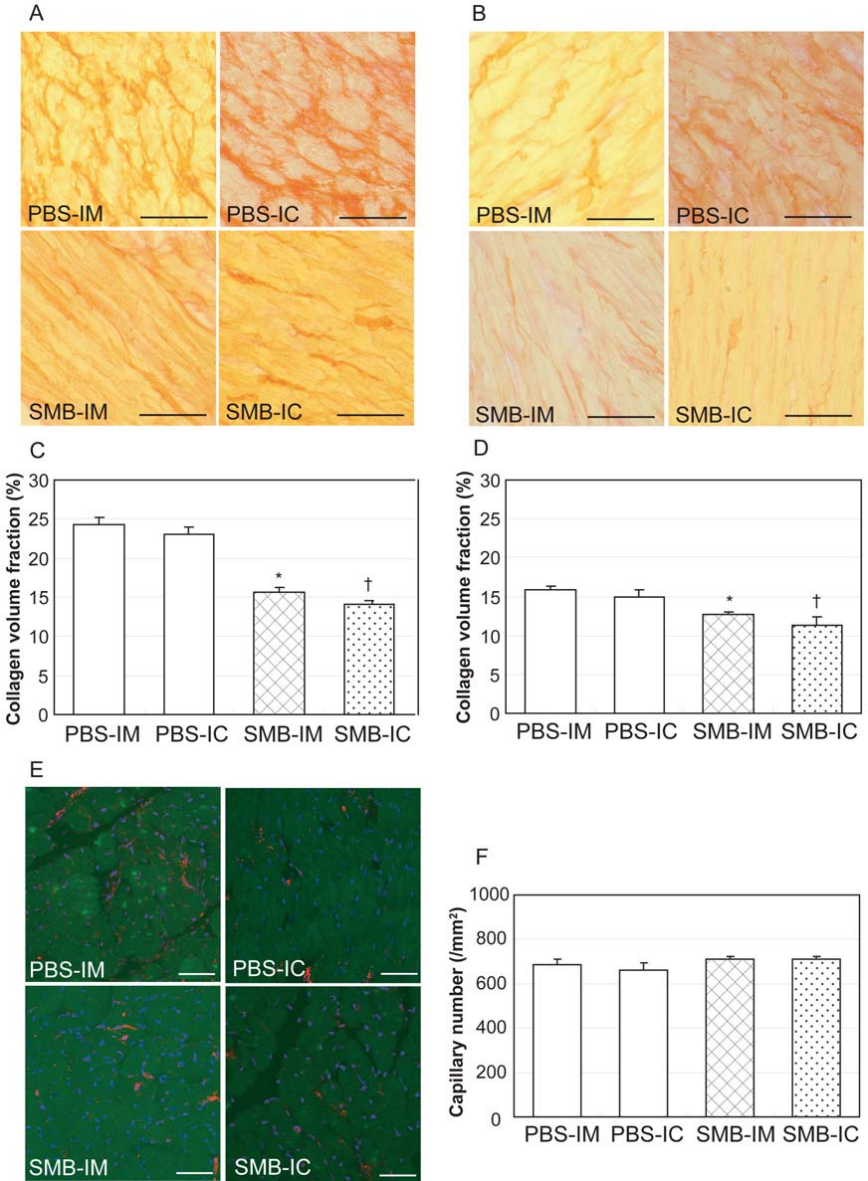
Extracellular collagen and capillary density in the myocardium. A: Extracellular collagen deposition in the myocardium was assessed by Picrosirus red staining at day 28. Representative pictures in the peri-infarct area show a marked accumulation of extracellular collagen (red colour) in the PBS-IM and PBS-IC groups, compared to the SMB-IM and SMB-IC groups (×400). B: Representative pictures of the infarct-remote area also show reduced collagen accumulation in the SMB-IM and SMB-IC groups, compared to the PBS-IM and PBS-IC groups (×400). C and D: Collagen volume in both the peri-infarct © and infarct-remote (D) areas, analysed by computer-assisted morphometry, was significantly smaller in the SMB-IM and SMB-IC groups, compared to the corresponding PBS groups. E: Capillary number at day 28, assessed by immunoconfocal microscopy (red for vWF, blue for nuclei), appeared to be similar among the groups. F: Capillary density, expressed as the number of capillary vessels per mm^2^, did not show a significant difference among any groups. Scale bar = 100 µm. *p<0.05 *vs.* the PBS-IM group; ^†^ *p*<0.05 *vs.* the PBS-IC group. *N* = 5 in each group.

Picrosirius red staining demonstrated that a marked collagen deposition occurred in the extracellular space in the peri-infarct areas after PBS injection with a collagen volume fraction of approximately 23% (Figure 6A and C). Fibrosis was comparably and significantly reduced in the myocardium after SMB injection *via* IM (15.6±0.6%) or retrograde IC (14.1±0.5%) route. Fibrosis was also observed in infarct-remote areas after PBS injection with the collagen volume fraction reaching approximately 15% (Figure 6B and D). This was also significantly decreased after SMB injection *via* IM (12.7±0.4%) or retrograde IC (11.3±1.2%) route. Capillary density, which was assessed in peri-infarct areas of vWF-labelled samples (Figure 6E), was not significantly different after SMB injection (710±13/mm^2^ in the SMB-IM group and 711±13/mm^2^ in the SMB-IC group), compared to the corresponding control group (685±24/mm^2^ in the PBS-IM group and 660±36/mm^2^ in the PBS-IC group, Figure 6F).

## Discussion

In the present study, we demonstrate that retrograde IC injection of SMB improves cardiac function of post-MI chronic HF. The therapeutic benefits were no less than that induced by IM injection of the same number of SMB. Cell-distribution was markedly different between IM and retrograde IC injection, but overall graft survival was very similar. Interestingly, we observed clear differences in the occurrence of arrhythmias dependent on the cell-delivery route used. Following IM injection of SMB, two different, time-dependent types of arrhythmia were seen; early-phase frequent VPC with spontaneous VT and following late-phase less-frequent VPC with latent VT. In contrast, after retrograde IC injection of SMB, such early-phase VPC or VT were absent. Regardless of the delivery-route, SMB injection into the post-MI heart reduced hypertrophy of native cardiomyocytes and decreased collagen deposition in both peri-infarct and infarct-remote areas, but did not affect vascular formation. Cardiomyogenic trans-differentiation of the grafted SMB or fusion of the grafted SMB with the native cardiomyocytes was only very rarely detected, suggesting an important role of paracrine effects in the observed therapeutic benefits of SMB transplantation.

The pattern of SMB-distribution was radically different between IM and retrograde IC injection. IM injection formed islet-like clusters of the grafted cells accompanied by myocardial inflammation, while retrograde IC injection achieved widespread SMB dissemination with little myocardial damage. Nevertheless, survival of the grafted cells, recovery of cardiac function, or improvement of physical activity after retrograde IC injection of SMB was, contrary to our expectation, not significantly greater than those after direct IM injection in the setting of our model. However, SMB transplantation *via* retrograde IC injection is, as compared to IM injection, likely to have greater potential to achieve further augmentation of the therapeutic efficacy by refining the injection protocol. Increasing injection pressure or extending incubation time after injection will enhance the efficiency of trans-endothelial migration of the grafted cells into the myocardial interstitium, which is considered to be a limiting process for successful cell-engraftment after retrograde IC injection but is not relevant to IM injection. In addition, the number of cells to be injected could be safely increased in retrograde IC injection, whereas increasing cell number in IM injection carries the risk of exacerbating inflammation and early-phase arrhythmogenicity. These improvements of the protocol for retrograde IC injection will be able to enhance the overall graft size and consequent therapeutic effects, though further investigations are needed to confirm this. Experiments using a large animal model would be appropriate to determine the optimal protocol of retrograde IC injection of SMB in clinical settings. In addition, more importantly, our study here demonstrated that retrograde IC injection dramatically attenuated arrhythmia occurrence in the early phase after injection, compared to IM injection. In the clinical arena, retrograde IC injection can be safely carried out by a balloon catheter percutaneously inserted into the cardiac veins [22]. This will be advantageous over the direct IM injection which requires either open-chest surgery or highly-specialised equipment such as the NOGA system [4]. Taking all this information into consideration, we propose that retrograde IC injection could be a more advantageous route for SMB transplantation for treating post-MI chronic HF compared to direct IM injection.

The present study provides important information to determine the mechanism by which SMB injection improves the function of post-MI chronically failing myocardium. Consistent with previous reports [5], we observed extremely rare events of cardiomyogenic differentiation of the grafted SMB or fusion with the native cardiomyocytes regardless of the cell-delivery route. Such an extremely low frequent event is unlikely to contribute for global cardiac function or arrhythmia occurrence regardless of its origin. Instead, there was significant regression of hypertrophy of the cardiomyocytes and reduction of pathological fibrosis not only in the peri-infarct but also in the infarct-remote areas, suggesting that paracrine effects may play an important role in the therapeutic benefits of SMB transplantation. Interestingly, unlike acute MI [10], neovascularisation was not enhanced by SMB injection in the present study targeting post-MI chronic HF, indicating that the paracrine effects may be affected by the condition of the native myocardium. Although Bonaros et al [23], [24] suggested angiogenic effects of SMB on post-infarction chronically failing heart, cell-injection timing and MI size are different from the present study. Differently-diseased myocardium could differently respond to the same paracrine stimuli, or might differently modulate the secretion from the grafted SMB.

LV dimensions and peak E/A ratio at 84 days following SMB injection *via* either route was not significantly greater than those following PBS injection, though LVEF and physical activity at 84 days following SMB injection were still significantly greater than those following PBS injection. We consider that LV dimensions and peak E/A ratio were not necessarily correlated with LVEF and physical activity, as each parameter was assessed by different methods individually. However, these findings could suggest transient effects of cell transplantation on post-MI heart. Further functional and pathological studies with a longer time period would clarify this issue.

Although arrhythmogenicity induced by SMB transplantation remains controversial [1], [3], [13], [25], [26], our study provided evidence for arrhythmia occurrence after SMB transplantation using a rat model of post-MI chronic HF which is free from anti-arrhythmic drugs. In the early period, IM injection, but not retrograde IC injection, of SMB produced a large number of spontaneous VPC including VT with a peak at day 3. This type of arrhythmias following IM injection largely disappeared by day 28 and was not observed following PBS injection. We consider that one of the causes of this early-phase arrhythmia is local heterogeneity, geographical and/or biological, generated in the post-MI failing myocardium. After IM injection, the grafted cells formed isolated, islet-like clusters in the peri-infarct area, which would cause serious disorder of the local myocardial structure and electrical conductance. In addition, these cell-clusters were associated with accumulation of CD45-positive inflammatory cells, which could amplify the disorder of myocardial organisation and might also affect intercellular communication and/or electrical stability of the surrounding native cardiomyocytes. These pathological events could lead to generation of ventricular arrhythmias. Retrograde IC injection, in contrast, disseminated donor-cells widely (without forming localised cell-clusters) with less inflammation and caused much less ventricular arrhythmias in the early phase, compared to IM injection. We recently reported that a similar early-phase ventricular arrhythmia is caused by IM injection of bone marrow mononuclear cells [6], suggesting that direct IM cell-injection may induce arrhythmogenicity in the early period regardless of the cell-type injected. Similar early-phase arrhythmias have also been observed in clinical trials of SMB transplantation [1]–[3], while it was also reported that prophylactic use of amiodarone [2] and corticosteroids [3], modifications of the protocol including the number of cells injected, number of injection sites/volume and use of human serum for SMB culture are effective in attenuating this type of arrhythmia [27], [28]. For future clinical application of the IM route for SMB transplantation, however, careful monitoring and appropriate treatment for the early-phase arrhythmia would be required.

In the late phase after SMB injection, we found a different type of arrhythmia occurrence from that in the early phase. There was a persistent, though not frequent, spontaneous VPC which was associated with a high risk of VT occurrence under stress as uncovered by isoproterenol-administration, regardless of the SMB-delivery route. This finding is consistent to the previous clinical observation of sustained VT for a long time after IM injection of SMB [1], [3]. We consider that the mechanism for this late-phase arrhythmia might be different from that in the early phase, as the local heterogeneity and inflammatory response observed in the early phase had largely decreased by this time point. Surviving grafted cells which acquire arrhythmogenic potential *via* trans-differentiation or fusion could be a focus of ventricular arrhythmias, although such events appeared to be extremely infrequent [25]. Surviving SMB-derived cells, despite a small number, might also be a cause of re-entrance arrhythmias. Or, the native cardiomyocytes might be adversely affected by paracrine effects of the grafts, leading to arrhythmogenicity. Importantly, such late-phase arrhythmias were not found following injection of bone marrow-derived cells *via* either route into the same animal model [6], indicating SMB-dependent mechanisms of late-phase arrhythmias. Further studies for determining the precise mechanism and developing prevention/treatment of the late-phase arrhythmogenicity following SMB transplantation would be important for the future clinical success of this therapy.

A possible limitation in our experimental model, in which SMB derived from GFP-transgenic male SD rat were injected into the female wild-type SD rat heart, was a host immune response against allogenicity, Y-chromosome and/or GFP. SD rat is not an inbred strain in a strict definition, but is very close to inbred. Actually, it has been successfully used for an organ or cell transplantation model for heart disease without use of immunodepressants [29], [30]. These reports have described little immune response-related inflammation, vasculopathy or organ dysfunction. Furthermore, GFP-transgenic animals have been frequently used for a variety of cell therapy models [31], which did not show any evidence of host-immune response against the GFP. Moreover, gender mismatched-cell therapy or organ transplantation has been carried out with few findings of the immune rejection [32], [33]. In addition, the results of our previous experiments using female immunodeficient rats as a host animal have demonstrated that such a risk is negligible in our experimental system. Histological findings, the degree of improvement in cardiac performance measured by echocardiography, and graft survival measured by real-time PCR for *Sry* following transplantation of SMB derived from male GFP-transgenic SD rats into female wild-type SD rats were identical to those when the same cells were injected to female immunodeficient rats (data not shown). These findings suggest that the host immune response against allogenicity, GFP or Y-chromosome does not induce inflammation or impair the fundamental behaviour of the grafted cells and consequent therapeutic outcomes.

In summary, we have demonstrated that retrograde IC injection of SMB was able to provide similar, or possibly greater, therapeutic benefits in treating ischaemic cardiomyopathy with less arrhythmogenicity in the early phase, compared to direct IM injection. This data demonstrates the promising utility of the retrograde IC route for SMB transplantation to treat post-MI chronic HF. Persistent arrhythmogenicity in the late phase remains a concern of SMB transplantation regardless of the delivery-route.
