# Correction: Choice of cell-delivery route for skeletal myoblast transplantation for treating post-infarction chronic heart failure in rat

**DOI:** 10.1371/journal.pone.0348622

**Published:** 2026-05-05

**Authors:** Satsuki Fukushima, Steven R. Coppen, Joon Lee, Kenichi Yamahara, Leanne E. Felkin, Cesare M. N. Terracciano, Paul J. R. Barton, Magdi H. Yacoub, Ken Suzuki

Following publication of this article [1], the following errors were identified in the Methods and Results sections of the article text and in Fig 6 and Table 1:

- In the Assessment of cardiac function and structure section of the Methods, there is an error in the group sample size reported in the first sentence. The correct sentence is: Echocardiography (Sequoia 512 and 15-MHz probe, Siemens Medical) was carried out under 1.5% isoflurane inhalation via a nose corn at one day before, and days 3, 7, 28 and 84 after injection (*n*  =  9 in each group at each time point) [6].
- There is an error in the group sample size reported in the first footnote of Table 1 of [1]. Please see the complete and correct Table 1 provided with this Correction.
- There is an error in the % mortality reported in the first sentence of the Mortality after LCA ligation and cell injection of the Results section. The correct sentence is: Mortality after LCA ligation before cell injection was 11.1% (21/190) in total.
- The PBS-IM panel in Fig 6B appears similar to Fig 2G of [2,3].

The authors note that the PBS-IM panel in Fig 6B of [1] and in the originally published version of Fig 2G of [2,3] are duplicate images that represent the same experimental conditions. Although reuse of the image in [2] was unintentional, the control group tissue was shared between concurrently-run projects to reduce the number of animals used. With this Correction, the authors provide the following additional text to the start of the Generation of post-MI chronic HF and SMB injection subsection of Methods:

*This study was concurrently conducted in conjunction with another project* [*6*] *using the same rat HF model to reduce the number of animals suffering substantial-severity procedures. For the common groups between the projects, including PBS-IM and PBS-IC groups, the tissue samples or research data, which were created and used in the other study [6], were reused as a part of those in this study.*

With this Correction, the authors provide additional methodological information as follows:

Animals were humanely killed by overdose of anesthesia (5% isoflurane inhalation until at least one minute after animal’s breathing stopped). Following the substantial-severity procedure, the health and behaviors of animals were closely monitored three times per day for the initial three days, twice per day between 4–7 days post-procedure, and once per day afterwards. The appearance, behavior, and activity of the rats, their consumption of water/food, and signs of local infection were examined. Body weight of rats was monitored every 2 days for the first week and once per week afterwards. Any premature animal death was reported to the Named Veterinarian and Institute’s Animal Ethics Committee through the Biological Sciences Unit. Twenty-one rats were found dead prior to implementing a humane end point, but those which were excluded due to having an LVEF greater than 40% were humanely euthanized.

With this Correction, the corresponding author shares the available image and quantitative data underlying Fig 6 (S1-S2 Files). They confirmed no other original data are available.

**Table 1:** Cardiac performance after SMB transplantation.

	Before				Day 28				Day 84			
	**HR (bpm)**	**LVDd (mm)**	**LVDs (mm)**	**Peak E/A**	**HR (bpm)**	**LVDd (mm)**	**LVDs (mm)**	**Peak E/A**	**HR (bpm)**	**LVDd (mm)**	**LVDs (mm)**	**Peak E/A**
PBS-IM	373 ± 20	8.8 ± 0.2	7.3 ± 0.2	1.3 ± 0.2	406 ± 12	10.0 ± 0.2	8.7 ± 0.2	0.7 ± 0.1	431 ± 43	9.7 ± 0.2	8.9 ± 0.4	0.8 ± 0.0
PBS-IC	370 ± 23	8.7 ± 0.1	7.4 ± 0.2	1.4 ± 0.2	401 ± 11	9.5 ± 0.2	8.8 ± 0.2	0.8 ± 0.1	432 ± 29	10.0 ± 0.4	8.7 ± 0.5	0.8 ± 0.1
SMB-IM	370 ± 10	9.0 ± 0.1	7.5 ± 0.1	1.3 ± 0.1	354 ± 9*	9.9 ± 0.2	8.0 ± 0.2*	1.8 ± 0.1*	372 ± 12	10.6 ± 0.4	9.0 ± 0.4	1.3 ± 0.2
SMB-IC	369 ± 9	9.1 ± 0.1	7.7 ± 0.1	1.3 ± 0.1	356 ± 7†	9.6 ± 0.2	7.7 + 0.3†	1.5 ± 0.3†	372 ± 7	10.4 ± 0.4	9.3 ± 0.6	0.9 ± 0.2

*n* = 9 in each group at each time point.

* *p* < 0.05 *vs.* PBS-IM.

† *p* < 0.05 *vs.* PBS-IC.

## Supporting information

S1 File Partial image data underlying Fig 6. Fig 6A PBS-IM panel; Fig 6B PBS-IM and SMB-IM panels.
(ZIP)

S2 File Partial quantitative data underlying Fig 6C-D, F. Data are available for a limited number of samples from before data collection was complete.
(XLS)
