# Supplementary figures and images for: Correction: Choice of cell-delivery route for skeletal myoblast transplantation for treating post-infarction chronic heart failure in rat
Source: PLoS One. 2026 May 5;21(5):e0348622. doi: 10.1371/journal.pone.0348622 (PMC13143070; doi:10.1371/journal.pone.0348622)

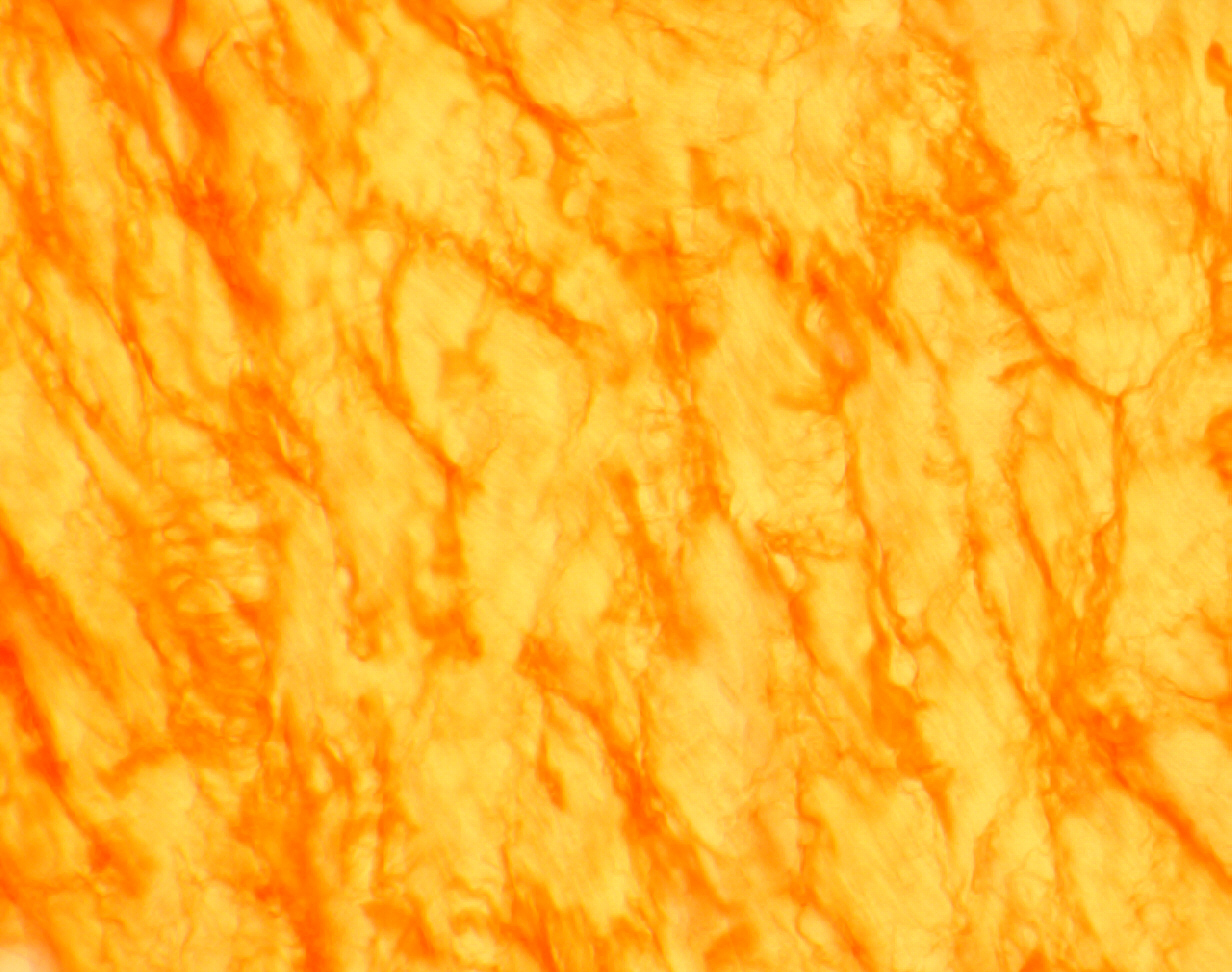

Supplement: S1 File — Fig 6A PBS-IM panel; Fig 6B PBS-IM and SMB-IM panels. (ZIP) [file pone.0348622.s001.zip › S1 File/Fig 6A PBS-IM.jpg]

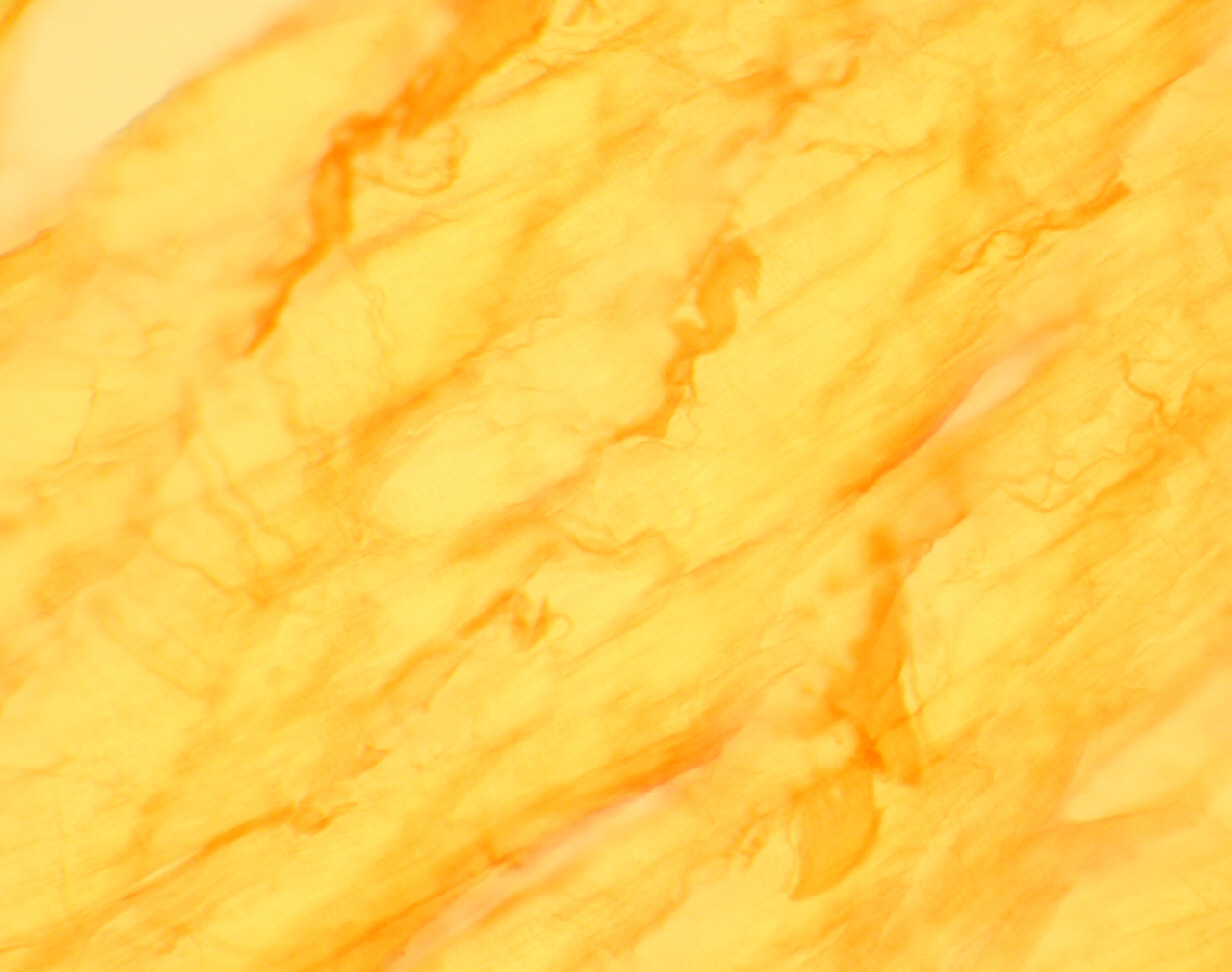

Supplement: S1 File — Fig 6A PBS-IM panel; Fig 6B PBS-IM and SMB-IM panels. (ZIP) [file pone.0348622.s001.zip › S1 File/Fig 6B PBS-IM.jpg]

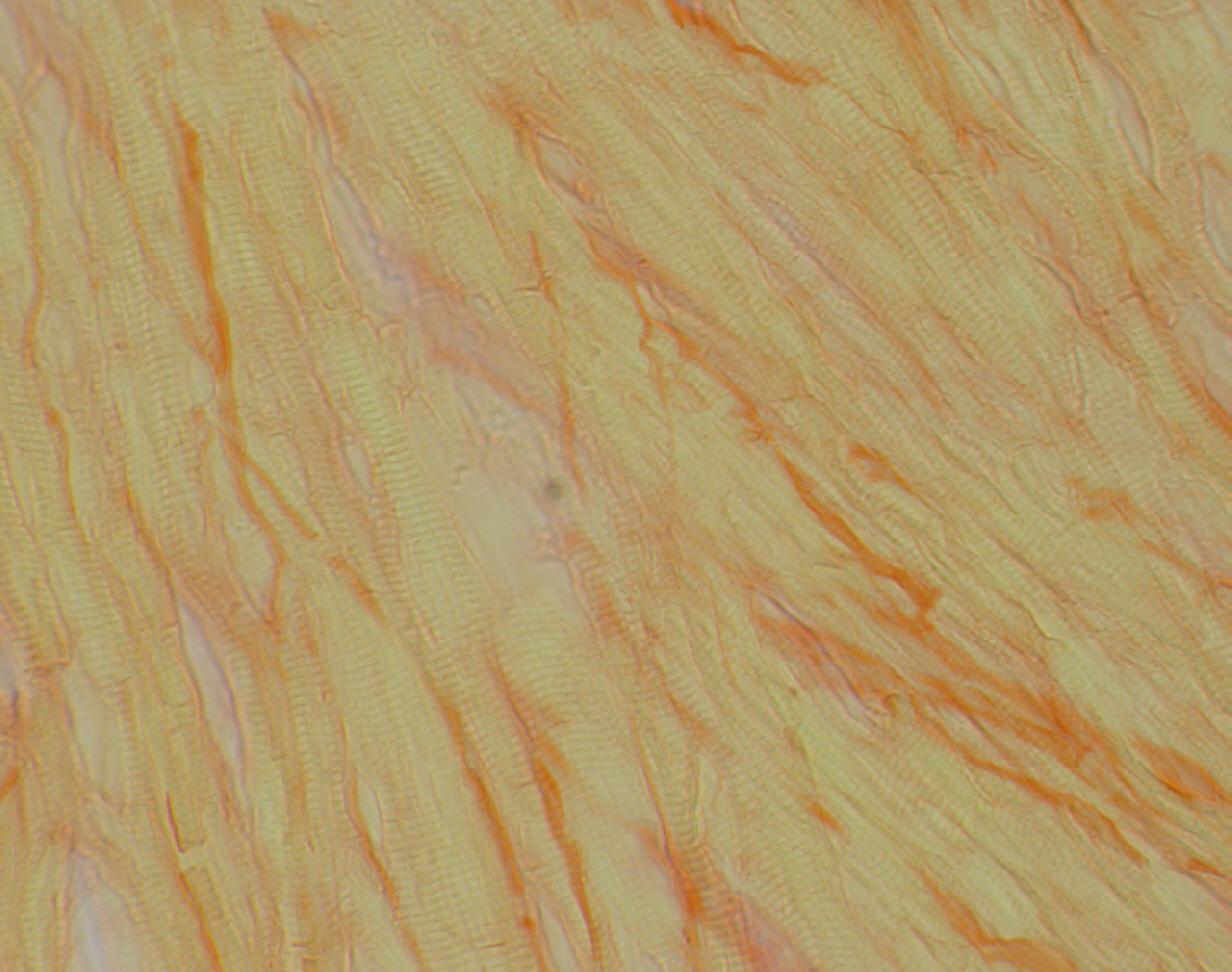

Supplement: S1 File — Fig 6A PBS-IM panel; Fig 6B PBS-IM and SMB-IM panels. (ZIP) [file pone.0348622.s001.zip › S1 File/Fig 6B SMB-IM.jpg]
